# A novel transgenic mouse model of CBS-deficient homocystinuria does not incur hepatic steatosis or fibrosis and exhibits a hypercoagulative phenotype that is ameliorated by betaine treatment

**DOI:** 10.1016/j.ymgme.2010.06.010

**Published:** 2010-10

**Authors:** Kenneth N. Maclean, Jakub Sikora, Viktor Kožich, Hua Jiang, Lori S. Greiner, Eva Kraus, Jakub Krijt, Katherine H. Overdier, Renata Collard, Gary L. Brodsky, Lynne Meltesen, Linda S. Crnic, Robert H. Allen, Sally P. Stabler, Milan Elleder, Rima Rozen, David Patterson, Jan P. Kraus

**Affiliations:** a Departments of Pediatrics and Medicine, University of Colorado School of Medicine, Aurora, CO, USA; b Institute of Inherited Metabolic Diseases, Charles University, 1st Faculty of Medicine, Prague, Czech Republic; c Departments of Human Genetics and Pediatrics, McGill University, Montreal Children's Hospital, Montreal, Quebec, Canada; d Eleanor Roosevelt Research Institute and the Department of Biological Sciences at the University of Denver, Denver, CO, USA

**Keywords:** BHMT, betaine-homocysteine *S*-methyltransferase, HCU, classical homocystinuria, CBS, cystathionine beta-synthase, CGL, cystathionine gamma-lyase, Hcy, homocysteine, MTHFR, methylenetetrahydrofolate reductase, AdoMet, *S*-adenosylmethionine, AdoHcy, *S*-adenosylhomocysteine, tHcy, total homocysteine, Betaine, Coagulation, Cystathionine, Cystathionine beta-synthase, Cystathionine gamma-lyase, Homocystinuria, Homocysteine

## Abstract

Cystathionine beta-synthase (CBS) catalyzes the condensation of homocysteine (Hcy) and serine to cystathionine, which is then hydrolyzed to cysteine by cystathionine gamma-lyase. Inactivation of CBS results in CBS-deficient homocystinuria more commonly referred to as classical homocystinuria, which, if untreated, results in mental retardation, thromboembolic complications, and a range of connective tissue disorders. The molecular mechanisms that underlie the pathology of this disease are poorly understood. We report here the generation of a new mouse model of classical homocystinuria in which the mouse *cbs* gene is inactivated and that exhibits low-level expression of the human *CBS* transgene under the control of the human *CBS* promoter. This mouse model, designated “human only” (HO), exhibits severe elevations in both plasma and tissue levels of Hcy, methionine, *S*-adenosylmethionine, and *S*-adenosylhomocysteine and a concomitant decrease in plasma and hepatic levels of cysteine. HO mice exhibit mild hepatopathy but, in contrast to previous models of classical homocystinuria, do not incur hepatic steatosis, fibrosis, or neonatal death with approximately 90% of HO mice living for at least 6 months. Tail bleeding determinations indicate that HO mice are in a hypercoagulative state that is significantly ameliorated by betaine treatment in a manner that recapitulates the disease as it occurs in humans. Our findings indicate that this mouse model will be a valuable tool in the study of pathogenesis in classical homocystinuria and the rational design of novel treatments.

## 1 Introduction

Cystathionine beta-synthase (CBS) (EC 4.2.1.22, CBS) catalyzes the pyridoxal 5′-phosphate (PLP) dependent condensation of serine and homocysteine (Hcy) to form cystathionine, which is then converted to cysteine by another PLP-dependent enzyme, cystathionine gamma-lyase (CGL). CBS deficiency is the most common cause of classical homocystinuria (HCU), an inherited autosomal recessive metabolic disease, which, if untreated, causes skeletal abnormalities, dislocated optic lenses, mental retardation, and a dramatically increased incidence of vascular disorders particularly thromboembolic disease [1].

The pathogenic mechanisms that underlie this disease are poorly understood. To date, the majority of animal studies of the disease have relied upon a previously described *cbs* null knockout mouse model that suffers from growth retardation and severe hepatopathy with 90% of *cbs* null mice dying during the first 2 weeks of neonatal life [2]. Surviving *cbs* null mice appear to incur severe hepatopathy and lung fibrosis that is not observed in the human disease ([3] and our accompanying paper). This semi-lethal phenotype severely limits the practical use of the mouse model and its use in mechanistic studies is complicated by both its failure to recapitulate key aspects of the human disease and the severe hepatopathy that has the potential to influence the results. Consequently, there is a pressing need for a suitable animal model of HCU that more accurately reflects the human disease.

We report here the generation of a novel transgenic mouse model of HCU that is null for the mouse *cbs* gene and that carries two copies of the human *CBS* gene that are expressed at low level. This “human only” (HO) mouse exhibits severe elevations in both plasma and tissue levels of total Hcy (tHcy), methionine, *S*-adenosylmethionine (AdoMet), and *S*-adenosylhomocysteine (AdoHcy) and a concomitant decrease in plasma and tissue levels of cysteine. In contrast to previous models of HCU, this model does not incur any hepatic steatosis, fibrosis, or neonatal death. Tail bleeding determinations indicate that HO mice are in a hypercoagulative state that is significantly ameliorated by betaine treatment in a manner that recapitulates the disease as it occurs in humans. Our findings indicate that this mouse model will be a valuable tool in the study of pathogenesis in HCU.

## 2 Materials and methods

### 2.1 Animal studies

A breeding pair of heterozygous C57BL/6 j-*cbs*^tm1unc^ *cbs* ^+/^^−^ mice was obtained from the Jackson laboratory and was subsequently backcrossed to C57BL/6 J mice (The Jackson Laboratory, Bar Harbor, ME) for at least seven generations. To differentiate between this previously described mouse model of CBS deficiency and the new model described in this paper, we refer to the previous mouse model as the “Maeda knockout” (MKO) strain. The generation of a transgenic mouse strain carrying two copies of the human *CBS* gene has been described previously [4]. The 11181 mouse derivative of that strain was selected for crossing with *cbs*^+/^^−^ as non-quantitative RT–PCR analysis of this strain showed evidence of transcription of the human *CBS* gene. All mice were maintained on standard chow (LabDietNIH5K67, PMI nutrition international, Brentwood, MO).

Mice containing both the human *CBS* transgene and no functional copy of the mouse equivalent gene were identified by PCR from the litters of progeny of 11181 × MKO^+/^^−^ F1 mice backcrossed to MKO^+/^^−^ mice. These mice were designated as “human only” (HO) mice. Methylenetetrahydrofolate reductase (MTHFR) ^−^^/^^−^ mice and wild type littermate controls were generated at McGill University (Montreal, Canada) and sampled for plasma as described previously [5]. All experiments were approved by the University of Colorado Health Sciences Center institutional animal care and use committee and were performed according to the NIH standards for animal care and use.

### 2.2 Genotype determinations

Genomic DNA for genotyping was prepared from tail biopsies using the Mouse Tail kit D-7000B from Puregene (Minneapolis, MN) according to the manufacturer's instructions. All PCRs were performed using Herculase polymerase (Stratagene) and a Stratagene Robocycler. PCRs designed to determine either the presence or absence of mouse *cbs* exon 3 or to test for the presence or absence of the neo gene replacing exons 3 in the heterozygous and homozygous knockout mice were performed as described in our accompanying paper. In addition, HO mouse genomic DNA was examined for the presence of the human *CBS* gene using oligonucleotide primers #382 5′-CCT ACT GTG TGT GTT CAT CC-3′ and # 383 5′-TCC TTG GCT TCC TTA TCC-3′, which correspond to sequence in intron 0 and exon 0 of the human gene, respectively, which is not conserved in the mouse *cbs* gene. This reaction was performed at 94 °C for 2 min followed by 30 cycles at 94 °C 30 s, 52 °C 30 s, 72 °C 2 min 30 s, followed by 5 min at 72 °C. All CBS-deficient mice had their genotype independently confirmed by determination of their tHcy and cystathionine levels in plasma samples acquired by nonlethal tail bleeding.

### 2.3 FISH analysis of mice

To determine the number and location of integration sites of the human P102D1 DNA construct carrying the human *CBS* gene in the transgenic mice and derivative strains, linearized P102D1 DNA was labeled with digoxygenin dUTP (Boehringer-Mannheim) by nick translation and hybridized to blood smears obtained from nonlethal tail bleeding. The P1 clone probe was detected with anti-digoxygenin antibodies [6].Chromosomes were counterstained with 4,6 diamidino-2-phenylindole (DAPI) in antifade solution. FISH analysis was also performed on the human *CBS* transgenic line 11181 with the mouse chromosome 17 telomeric specific probe 17T (BAC50F18) [7] to ensure that the inserted human *CBS* gene is not located on chromosome 17 with the mouse *cbs* gene.

### 2.4 RT–PCR analysis of mice

Total RNA was isolated from the tissues indicated using guanidinium thiocyanate [8]. One microgram of total RNA was used for first strand synthesis using random hexamers with MuLV reverse transcriptase following instructions supplied by the manufacturer (Perkin-Elmer). PCR was performed using primers specific for human *CBS* exon 0 as described above. The identity of the amplified 581-bp band was confirmed by direct DNA sequencing.

### 2.5 Thiols and methionine cycle metabolites

Determination of plasma levels of amino acids and AdoMet and AdoHcy was performed as described previously [9, 10]. In mouse tissues, the levels of free aminothiols (i.e., nonprotein bound aminothiols) were determined as described in our accompanying paper.

### 2.6 Histological examination of mouse tissues

Mice were sacrificed by decapitation, and selected tissues were immersion-fixed overnight in 4% paraformaldehyde in PBS (pH 7.3). Paraffin-embedded sections were stained for examination with hematoxylin and eosin. For ultrastructural studies, parallel samples of livers were postfixed in 1% phosphate-buffered (pH 7.4) OsO_4_, dehydrated in ethanol, embedded in Epon-araldit with uranyl acetate and lead hydroxide, and viewed on a Tesla 500 electron microscope (Tesla, Czech Republic).

### 2.7 SDS–PAGE and Western blotting

Liver samples were homogenized in buffer containing 100 mM KPi, pH 7.4, 1 mM EDTA, and 1:50 (vol./vol.) protease inhibitor cocktail from Sigma. The ratio of liver tissue to lysis buffer was 1 g of liver tissue to 5 ml of lysis buffer. The homogenate was subsequently centrifuged at 4 °C at 20,000 × *g* for 20 min. The supernatant thus formed was used as a crude extract. The protein concentration of crude extracts was determined by the Bradford method using bovine serum albumin as a standard [11].

Denatured proteins were separated by SDS–PAGE using a 9% separating gel with a 4% stacking gel under reducing conditions [12]. Proteins were then transferred onto PVDF membrane using a semi-dry transfer cell (Bio-Rad). Resulting blots were probed with primary antibodies to CGL (H00001491-M02; Abnova used at 1:2500 vol./vol. dilution) and glyceraldehyde 3-phosphate dehydrogenase (GAPDH) (G9545; Sigma used at a 1:50,000 vol./vol. dilution). Signals were detected using a Typhoon 9400 system (Amersham Pharmacia) after incubation with the appropriate Fluorescein- or Texas red-conjugated secondary antibodies (Vector Laboratories) or Alexa Fluor 647-conjugated secondary antibody (Invitrogen). The relative intensities of protein bands were quantified using Quantity One version 4.6.5 software (Bio Rad). The signal intensity from CGL bands was calculated relative to signal intensity from GAPDH in liver.

### 2.8 Enzyme activity assays

For enzyme assays, all tissues were rapidly removed from the animal and flash frozen in liquid nitrogen. For determining CBS enzyme activities in mouse tissues 100–200 mg of tissue was homogenized in eight volumes of a buffer containing 30 mM potassium phosphate, pH 6.0, 1 mM beta-mercaptoethanol and 1:50 (vol./vol.) protease inhibitor cocktail (Sigma, P8340). The homogenate was centrifuged at 20,000 × *g* for 10 min and 20 μl of the supernatant thus formed was assayed in a total volume of 0.1 ml for 2 h at 37 °C as described previously [13] without the presence of exogenous AdoMet. One unit of CBS activity catalyzes the formation of 1 μmol of cystathionine in 1 h at 37 °C.

CGL enzyme activity was measured in liver extracts using a modification of a previously described enzyme-coupled assay with lactic dehydrogenase (LDH) [14]. Briefly, assay reactions containing 100 mM KPi, pH 8.0, 2 mM l-cystathionine, 175 mM NADH, and 5 U of LDH were preincubated to 37 °C. Reactions were started by the addition of 25 μg of crude liver extract to give a final reaction volume of 100 μl. The oxidation rate of NADH was monitored at 340 nm for 2 min at 37 °C as an index of CGL activity.

### 2.9 Assessment of coagulation parameters

The coagulative phenotype of *cbs* (−/−) mice was assessed by determination of tail bleeding times using a previously reported method as a surrogate of hemostasis and thrombosis function [8].

### 2.10 Statistical analysis

The Kaplan–Meier survival curves were constructed using Prophet 5.0, the differences in survival functions were tested by Mantel–Cox test using the same software. All tests were Bonferonni-corrected where appropriate, significance level for tests was set at *P* = 0.05. For the tail bleeding time experiments, Kolmogorov–Smirnov tests indicated that the data were not normally distributed, and subsequent statistical analyses were performed using the nonparametric Kruskal–Wallis test. All other data were presented as means ± SD and were compared using the unpaired Student's *t* test. A *P* value of less than 0.05 was considered statistically significant. In the graphed data, *, **, and *** denote *P* values of *<* 0.05, 0.01, and 0.001, respectively.

## 3 Results

### 3.1 Expression of the human *CBS* gene does not significantly alter homocysteine metabolism in the human *CBS* transgenic mouse line 11181

**Fig. 1:**
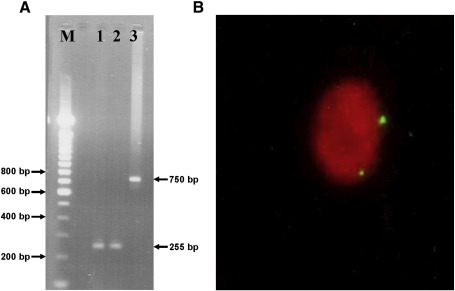
Characterization of the human *CBS* transgenic mouse 11181. (A) PCR analysis of human *CBS* gene expression. M, 100 bp ladder size marker (Invitrogen); 1, transgenic mouse line 11181 RT–PCR using primers in human exon 1 #187 5′-CGGAACACCAGGATCCCATGAC-3′ and #145 5′-GGTGCACCTGCTCGGAGCAT-3′ to yield a 255-bp fragment; 2, human *CBS* cDNA control; 3, human *CBS* fragment amplified from 11181 genomic DNA using primer 5′-CGTTCGAGAGTCCCAGGTG-3′ and 5′-TCC TTG GCT TCC TTA TCC-3′. (B) FISH analysis showing two copies of the human *CBS* gene chromosomally integrated in 11181 mouse nucleus.

**Fig. 2:**
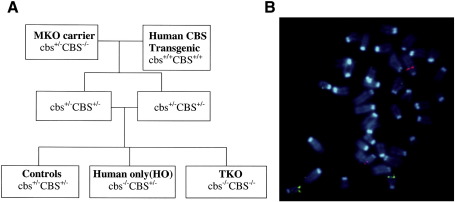
(A) Breeding scheme used to generate HO mice containing the human *CBS* gene but no functional mouse *cbs* gene. (B) FISH analysis of metaphase chromosomal spreads from HO *CBS* transgenic mice. 60.4P102D1 DNA (red) and T17 DNA (green) [4] were used to detect the human *CBS* transgene and the subtelomeric region of mouse chromosome 17, respectively. HO transgenic mouse chromosomes were hybridized with both probes simultaneously. The single copy of the human *CBS* transgene is not on mouse chromosome 17 where the mouse *cbs* gene is located [28].

Human *CBS* transgenic mouse lines carrying the entire human *CBS* gene and approximately 50 kb of 5′ upstream sequence in addition to two copies of the equivalent mouse *cbs* gene have been described previously [4]. Initially, it was unknown as to whether the human *CBS* gene is expressed in these lines, and early initial screening was performed using an RT–PCR designed to specifically amplify human CBS mRNA (Fig. 1A). On this basis, we selected one transgenic mouse line, designated 11181, and selectively crossed them to generate mice carrying two copies of the human *CBS* gene in addition to the normal two copies of the mouse *cbs* gene (Fig. 1B). Preliminary analysis of these mice showed no significant difference in basal levels of plasma total Hcy (tHcy), cystathionine, cysteine, or methionine compared to normal controls, indicating that the extra copies of the human *CBS* gene were not adding significantly to the total level of CBS activity (data not shown). To explore this further, we performed a series of methionine loading experiments using the 11181 mice and normal controls. In these experiments, control mice and 11181 mice (*n* = 6) showed baseline plasma levels of tHcy of 6.01 μM (SD = 1.3) and 5.6 μM (SD = 1.6), respectively, which were not statistically significantly different from each other (*P* = 0.6367). Maximal tHcy was observed at 3 h after methionine loading with control and 11181 mice exhibiting plasma tHcy levels of 28.0 μM (SD = 3.9) and 30.05 μM (SD = 4.3), respectively, which did not differ significantly from each other (*P* = 0.4073). At 6 h after loading, the plasma levels of tHcy of both groups of mice had returned to a value that was not statistically significantly different from their respective baselines. Collectively, these results indicate that the 11181 mice show no significant difference in their ability to process excess Hcy. These findings presented us with two possible interpretations. Either the human *CBS* gene is not being expressed at a significant level in the 11181 mice or both the human *CBS* and mouse *cbs* genes are being expressed at significant levels but homeostatic mechanisms exist designed to restrict total CBS expression within defined limits. This latter possibility is very difficult to investigate in 11181 mice because the mouse and human CBS proteins are virtually identical in terms of molecular mass and are indistinguishable by SDS–PAGE. To address this question, we bred 11181 mice bearing two copies of the human *CBS* gene with MKO heterozygous *cbs*^+/^^−^ mice in a series of crosses outlined in Fig. 2A. These crosses were designed to generate mice that contain only the human *CBS* gene so that the expression level of this gene could be examined in isolation. The litters containing these mice designated “human only” (HO), also contained hybrid *cbs* knockout mice with no functional copy of the mouse *cbs* or the human *CBS* genes. These latter mice were designated as transgenic knockouts (TKO) to distinguish them from the previous *cbs* (−/−) null knockout model generated previously by Dr Noboru Maeda et al. [2]. Fluorescent in situ hybridization (FISH) analysis was used to determine the number of insertion sites of the human *CBS* gene that were integrated into the mouse genome in the HO mouse and to confirm that the human gene has integrated on a different chromosome from the mouse gene (Fig. 2B).

### 3.2 The human *CBS* gene is expressed at very low levels in HO mice resulting in a biochemical phenotype consistent with HCU

**Fig. 3:**
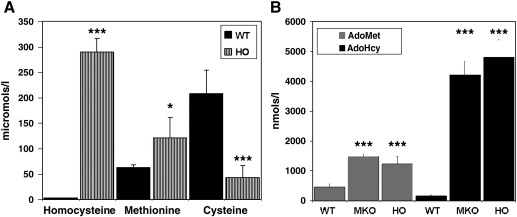
HO mice exhibit a biochemical profile consistent with HCU. Plasma levels of (A) tHcy, methionine, and total cysteine in wild type mice and HO mice. Values shown represent the mean and SD derived from a minimum of 10 animals. (B) Mean and SD of plasma AdoMet and AdoHcy in WT controls (*n* = 10) MKO *cbs* null mice (*n* = 5) and HO mice (*n* = 10). In this figure and all subsequent graphs presented here *, **, and *** denote *P* values of *<* 0.05, 0.01, and 0.001, respectively.

In our initial characterization of the HO mouse model, we determined the plasma levels of tHcy, methionine, total cysteine, AdoMet, and AdoHcy in 18 HO mice. This analysis (Figs. 3A and B) found that the HO mice exhibited a biochemical phenotype consistent with HCU. Plasma levels of tHcy, methionine, AdoMet, and AdoHcy were elevated 83-fold, 2-fold, 2.6-fold, and 29-fold, respectively, compared to control wild type mice (*n* = 20, *P* < 0.0001 for all four metabolites). Conversely, plasma cysteine levels in HO mice were decreased approximately 5-fold (*P* < 0.0001) compared to controls.

In addition to this plasma analysis, we also determined the levels of free Hcy, cysteine and glutathione in liver, brain, kidney, heart, and calf muscle of HO mice (*n* = 5). The levels of free Hcy, cysteine, and glutathione in these tissues were essentially identical (no statistically significant difference for any of these metabolites between the two models, *P* > 0.05) to those reported for the MKO *cbs* (−/−) null mice described in our accompanying paper (data not shown).

**Fig. 4:**
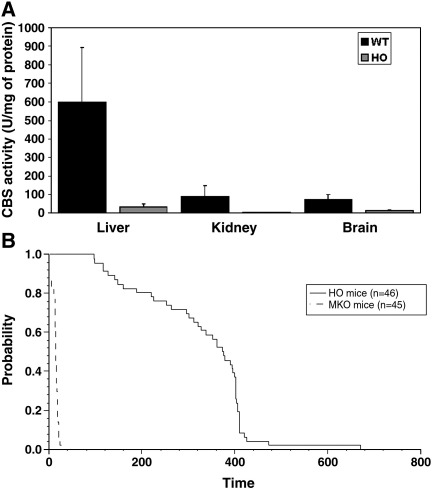
Low-level expression of the human *CBS* gene in HO mouse results in significantly improved survival compared to *cbs* null mice. (A) CBS activity in liver, brain, and kidney of HO and wild type control mice. Values shown represent the mean and SD derived from *n* = 3–7 for each tissue. CBS activity was assayed as described previously [13]. (B) Kaplan–Meier analysis showing a significantly improved survival for HO mice compared to MKO *cbs* null mice (*P* = 0.001). The *x* axis denotes survival time given in days post partum.

Collectively, the plasma and tissue amino acid levels in the HO mouse are consistent with either ablated or very low-level expression of the human *CBS* transgene, and these mice effectively constitute a novel mouse model of HCU. To confirm this interpretation, we determined the relative levels of CBS activity in tissue extracts derived from wild type control and HO mouse liver, kidney, and brain (*n* = 3–7 for both groups) as described in the Materials and methods section. We observed very low levels of CBS expression in the HO-derived tissues with liver, kidney, and brain exhibiting 5% (*P* = 0.003), 2% (*P* < 0.0001), and 16% (*P* = 0.0005) of the expression level observed in the corresponding control mouse tissues, respectively (Fig. 4A). The low level of CBS expression, especially in the liver where the bulk of transsulfuration typically occurs, and the near absence of CBS expression in the kidney provide an explanation for the biochemical phenotype observed in the HO mice.

### 3.3 The normal tissue distribution of CBS expression is conserved in HO mice

Although CBS is known to be expressed in liver, kidney, and brain, this gene is not ubiquitously expressed in mammals and has a relatively limited tissue distribution [1]. In the HO mouse model, the human *CBS* gene is expressed under the control of the human promoter, and it is unknown if these sequences are sufficient to maintain the normal pattern of tissue-specific expression in mice. To address this question, we also determined CBS enzyme activity levels in lung and muscle tissue homogenates from both HO and control mice that are normally devoid of CBS activity. We were unable to observe any CBS catalytic activity in either of these tissues in either the HO or wild type mice indicating that although expression of the human *CBS* gene in the HO mouse model is very much decreased, the normal pattern of tissue expression appears to be conserved (data not shown).

### 3.4 The HO mouse model of HCU does not exhibit neonatal lethality

One of the major problems with using the previously described MKO *cbs* null mouse model is the high level of neonatal lethality that is incurred by *cbs* (−/−) mice where approximately 90% of mice die within 2 weeks. The breeding strategy used to generate HO mice also generates TKO littermate mice that are also null for both mouse and human *CBS*. TKO mice were detected only occasionally, and these mice also typically died during the early neonatal period. Liver samples from surviving TKO mice showed profound hepatopathy, and plasma amino acid profiles in terms of tHcy, cystathionine, methionine, and cysteine were essentially identical to those observed in the MKO *cbs* null mice and were not studied any further.

We performed a Kaplan–Meier analysis of survival times of the HO mouse model and compared it to the MKO *cbs* null mice. It can be seen (Fig. 4B) that the HO mice exhibit a highly significant improvement in terms of survival and longevity compared to the MKO *cbs* (−/−) mice (*P* = 0.001). Significantly, no neonatal death was observed, and all HO mice observed lived for at least 80 days. Greater than 50% of the HO mice were alive at 1 year, indicating that the HO mouse model has sufficient longevity to facilitate detailed investigation of pathogenesis in HCU.

### 3.5 HO mice do not exhibit hepatic steatosis or fibrosis

**Fig. 5:**
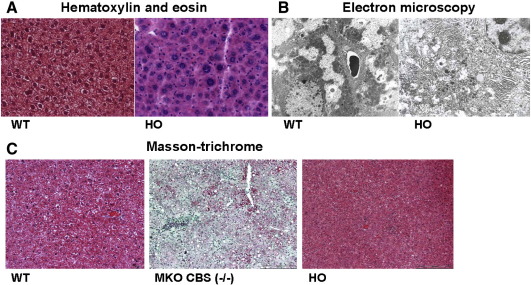
The HO mouse model of HCU does not exhibit steatosis or fibrosis. (A) Hematoxylin and eosin staining of HO and control mouse liver. HO mouse livers exhibit enlarged, binucleated cells, intranuclear pseudoinclusions, chromatin clumping, and enlarged nucleoli. Overall, these mice present with a mild hepatopathy dominated by a nuclear anisokoria and signs of hyperregeneration without any histologically detectable steatosis or fibrosis. (B) Electron microscopy of HO and control mouse liver (×8000). HO mice exhibited increased numbers of mitochondria and rough ER without significant dilation. No steatosis was observed. (C) Masson trichrome staining of HO mice. No fibrosis was observed in any HO (right panel) or wild type control (left panel) mouse samples. Liver samples from moribund MKO *cbs* null mice (middle panel) were used as a positive control for staining. Photomicrographs shown are representative of staining from 20 views taken from five HO mice and five wild type controls and were assessed by a pathologist without knowledge of genotype. Scale bars, 200 μm.

In terms of plasma and tissue levels of tHcy, methionine, and cysteine, AdoMet, and AdoHcy, the HO mouse model has an essentially identical biochemical profile to that observed in the previously described MKO *cbs* null mouse model (see accompanying paper). Despite this, the HO mice do not incur any neonatal lethality. In our accompanying paper, we present data indicating that the neonatal lethality observed in the MKO *cbs* null model is due to severe hepatopathy leading to liver failure. To investigate the possible link between neonatal lethality and hepatopathy in mouse models of HCU, we sacrificed 10 HO mice and examined their livers for evidence of dysfunction. In contrast to livers isolated from surviving MKO *cbs* null mice, the livers from HO mice were not enlarged and exhibited normal coloration. Histological examination using hematoxylin and eosin staining and ultrastructural examination using electron microscopy indicated that HO mice share similar signs of hepatopathy in terms of hepatocyte alteration as that observed in the MKO *cbs* ^−^^/^^−^ mice (nuclear anisokoria and signs of hyperregeneration), but crucially, there was no detectable steatosis or evidence of ER damage in any of the HO mice examined (Figs. 5A and B). Masson trichrome staining was used to assess the livers for possible fibrosis. No abnormal collagen staining was observed in any of the HO livers examined (Fig. 5c). These observations indicate that HO mouse livers are significantly less affected than the MKO null mouse model and are consistent with a causal link between hepatopathy and longevity in mouse models of HCU that is independent of tHcy levels.

### 3.6 The HO mouse responds biochemically to betaine

**Fig. 6:**
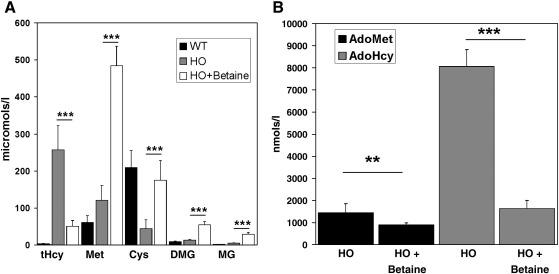
HO mice respond biochemically to betaine treatment. (A) Comparison of plasma tHcy, methionine (Met), total cysteine (Cys), dimethylglycine (DMG), methylglycine (MG) in WT controls, and HO mice plus and minus betaine. (B) Plasma AdoMet and AdoHcy in HO mice in the presence and absence of 1 week of betaine treatment (2% (vol./vol.) in drinking water given *ad libitum*). Values shown represent the mean and SD derived from 10 mice per group.

In the accompanying paper, we found that betaine treatment did not significantly lower plasma tHcy in MKO *cbs* (−/−) mice. We proposed that this was due to the influence of hepatopathy on betaine-homocysteine *S*-methyltransferase (BHMT) expression levels in these mice. As the HO mice present with a much milder level of hepatopathy, we investigated if betaine treatment would lower plasma tHcy levels in this model of HCU. We collected plasma samples from 10 HO mice before and after 1 week of betaine treatment as described in the Materials and methods section. A group of wild type mice (*n* = 7) without betaine treatment was also sampled for comparative purposes. The plasma levels of tHcy, the product of Hcy remethylation methionine, cysteine, and the betaine degradation products dimethylglycine (DMG) and methylglycine (MG) were then determined for all three groups (Fig. 6A). The HO mice showed a highly significant lowering of average tHcy levels from 257 (SD = 65) to 50 μM (SD = 16.1; *P* < 0.001) as a consequence of betaine treatment (Fig. 6A). Similarly, there was a 4-, 4.5-, 5.6-, and 4.7-fold increase in plasma methionine, DMG, MG, and cysteine, respectively (*P* < 0.0001 for all four metabolites). Lowering plasma tHcy by betaine treatment resulted in a 40% decrease in plasma AdoMet (*P* = 0.0039; Fig. 6B). Betaine treatment of HO mice also resulted in a 5-fold decrease in AdoHcy levels (*P* < 0.0001). Collectively, these data indicate that the HO mouse recapitulates the biochemical response of human subjects with this disease to betaine treatment and thus constitutes a suitable model for future investigations of ways to optimize the therapeutic effects of this treatment in HCU.

### 3.7 The HO mouse exhibits elevated plasma cystathionine

**Fig. 7:**
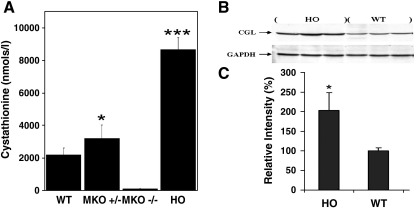
Despite having very low levels of CBS activity, HO mice exhibit significantly higher plasma levels of cystathionine compared to wild type control mice. The apparent accumulation of cystathionine in HO mice is not due to decreased expression of CGL (A) Plasma levels of cystathionine in wild type, MKO heterozygous (+/−), MKO null *cbs* (−/−), and HO mice. Values shown represent the mean and SD derived from a minimum of six mice. (B) Immunoblot analysis of hepatic CGL expression levels in HO and wild type control mice. Blotting and Immunostaining conditions were as described in the Materials and methods section. (C) The relative intensities of protein bands were quantified using Quantity One version 4.6.5 software (Bio Rad). Signal intensity from CGL bands was calculated relative to signal intensity from GAPDH in liver. The blot shown is representative of three independent experiments.

In an effort to elucidate why the HO mice present with only mild hepatopathy, we performed a detailed analysis of plasma metabolites relevant to transsulfuration in this model and compared it to that of the MKO *cbs* null mouse model. There was no significant difference in the plasma levels of tHcy, methionine, cysteine, serine, glycine, alpha-aminobutyrate, methyl- and dimethylglycine between MKO *cbs* −/− and HO mice (data not shown). The only metabolite to show any significant difference between these mouse models of HCU was cystathionine. This compound was completely absent from the MKO and TKO *cbs* null mice but was present at 4-fold higher than normal levels in the plasma of the HO mice (*P* < 0.0001; Fig. 7A). Although significantly elevated, the level of cystathionine observed in HO mice is lower than that observed in human subjects with cystathioninuria due to mutation in CGL. As cystathioninuria is essentially a benign condition, it is unlikely that the observed elevated cystathionine is exerting adverse effects in the HO mice [15].

### 3.8 Cystathionine accumulates in HO mice due to impairment of CGL function

**Fig. 8:**
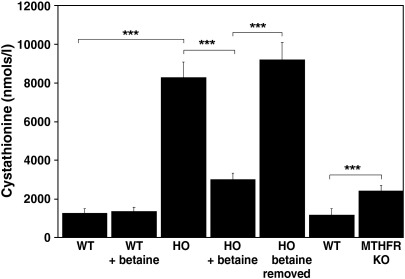
Plasma cystathionine levels are significantly lowered in HO mice by the Hcy reducing agent betaine. Cystathionine levels are elevated in MTHFR null (−/−) mice compared to wild type controls. Plasma samples were taken by non-lethal tail bleeding from 10 HO mice. Those mice were then treated with betaine for 1 week, and plasma samples were then retaken. Betaine treatment was removed for 1 week and the mice were sampled for plasma for a third time. The cystathionine levels were then determined from all samples. Wild type mice in the presence and absence of betaine treatment were used as controls. Basal plasma cystathionine levels were also determined for MTHFR null mice and their wild type littermate controls (*n* = 5 for each group). Values shown represent the mean and SD.

To our knowledge, transsulfuration is the only known endogenous source of cystathionine in mammals, and our work with the MKO *cbs* null mice has shown that the diet does not include any significant trace of this compound. This raises the question as to how the HO mice, with very low levels of CBS activity, can exhibit higher than normal levels of plasma cystathionine? As the level of CBS in HO mouse tissues effectively precludes elevated production of cystathionine, we hypothesized that the elevated cystathionine most likely reflected an accumulation of this compound due to impairment of the next enzyme in the pathway, CGL. To investigate this possibility, we used Western blotting analysis to determine the relative hepatic levels of CGL in HO mice compared to WT controls (Fig. 7B). The expression level of CGL was significantly higher in the HO mice than in the control mice indicating that the observed high levels of cystathionine are not due to transcriptional repression of CGL in the HO mice. This finding prompted an investigation into the effects of elevated Hcy upon CGL mediated conversion of cystathionine into cysteine at the protein level. CGL is a PLP-dependent enzyme and Hcy has the potential to impair CGL function by reacting with this co-factor. Preliminary studies using purified recombinant CGL enzyme indicated that high levels of Hcy act to reversibly inhibit CGL function and that this could be prevented by the addition of exogenous PLP (Jiang and Maclean, unpublished data). In the light of these preliminary data, we hypothesized that if Hcy were to be impairing CGL activity at the protein level, then lowering tHcy with betaine treatment should also serve to lower plasma cystathionine levels in HO mice. To test this hypothesis, we determined plasma tHcy and cystathionine levels in a group of HO mice (*n* = 10) first on water then after 1 week of betaine treatment and subsequently 1 week after the removal of betaine. We observed that lowering Hcy levels 5-fold by betaine treatment was accompanied by an approximate 3-fold lowering of plasma cystathionine levels in HO mice (*P* < 0.0001). Removal of betaine treatment resulted in both tHcy and cystathionine plasma levels returning to their pretreatment levels. Betaine treatment had no significant effect on plasma cystathionine levels in WT mice (*P* = 0.4311; Fig. 8).

Elevated cystathionine has been reported previously in other conditions that involve elevated tHcy [9, 16]. To further investigate our hypothesis that elevated Hcy causes cystathionine accumulation by impairing CGL function, we determined the cystathionine levels in plasma derived from methylenetetrahydrofolate reductase (MTHFR) null mice and their wild type littermate controls. In this analysis, we observed a significant increase in plasma tHcy compared to controls (mean of 35.75 μM, SD = 10.2 vs a mean of 5.7 μM, SD = 0.648, *P* = 0.00011, respectively) and a highly significant (*P* < 0.0001) increase in plasma cystathionine levels compared to the normal control mice (Fig. 8). Taken together, our findings indicate that elevated levels of Hcy act to impair the activity of CGL in HO mice and thus allow relatively high levels of cystathionine to accumulate despite severely impaired production of this compound in HO mice.

### 3.9 The HO mouse exhibits a hypercoagulative phenotype that responds to betaine treatment

**Fig. 9:**
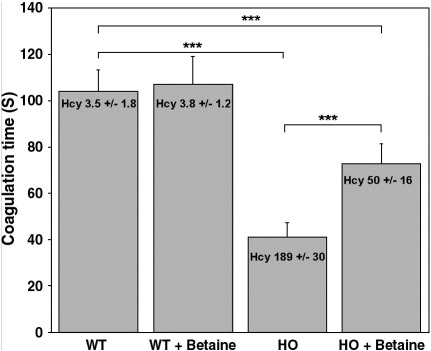
HO mice exhibit a hypercoagulative phenotype that responds to treatment with betaine. We determined tail bleeding times for the HO and wild type control mice as a surrogate of hemostasis and thrombosis function as described previously [8]. HO mice clotted approximately three times faster than the wild type controls (*P* = 0.0004) indicating that these mice are in a hypercoagulative state. Treatment of HO mice with betaine for 1 week significantly lowered the average value for tHcy and resulted in a significant increase in the average tail bleeding time, indicating that this treatment acts to ameliorate the hypercoagulative phenotype of the HO mouse model.

In our previous analysis of the *cbs* null mouse model, we observed no significant alteration of coagulation parameters. We hypothesized that this might be due to the severe hepatopathy observed in this model possibly offsetting any prothrombotic effects induced by elevated Hcy. As the HO mouse exhibits a much milder level of hepatopathy, we proposed to investigate if the HO mouse recapitulates the propensity for thrombosis typically observed in human subjects with HCU [1]. We determined tail bleeding times for the HO and wild type control mice as a surrogate of hemostasis and thrombosis function as described previously [8]. In these experiments, we found that the HO mice clotted approximately three-fold faster than the wild type controls (Fig. 9) (*P* = 0.0004) indicating that these mice are in a hypercoagulative state.

In human subjects with HCU, the combination of betaine treatment and a low methionine diet has proved to be effective in the prevention of thromboembolic complications. [1]. To investigate if betaine treatment could ameliorate the hypercoagulative phenotype exhibited by the HO mice, we determined the tail bleeding times of additional groups of HO and WT mice (*n* = 10 for each group) after treatment for 1 week with the Hcy lowering agent betaine. We observed that the betaine treatment significantly decreased plasma tHcy levels (*P* < 0.0001) and concomitantly increased the clotting time in the HO mice (*P* = 0.0005). This treatment had no significant effect upon either the plasma tHcy level (*P* = 0.7201) or clotting time in the WT control mice (*P* = 0.3245).

## 4 Discussion

The absence of severe hepatopathy and neonatal death in the HO mouse model of HCU is highly advantageous. What is presently unclear is why mice with CBS deficiency incur a level of hepatopathy that is not found in human subjects with this disease. In terms of their biochemical phenotype both the MKO and HO, mouse models exhibit a reasonably accurate model of the human disease. However, there appear to be significant differences between the relative elevations in methionine and AdoHcy induced by HCU in mice and humans that have the potential to contribute to hepatopathy in mice [17].

In addition to serving as an amino acid in protein synthesis, methionine also serves as a lipotrope in the liver and, as such, has the potential to ameliorate hepatic steatosis by facilitating the export of lipid from hepatocytes. It is conceivable that the greater tendency towards steatosis in *cbs* null mice compared to humans with this disease is a reflection of their having the same levels of Hcy but lower levels of methionine. Alternatively, the very high levels of AdoHcy found in the MKO mouse model of HCU result in an AdoMet/AdoHcy ratio of approximately 0.35 representing a highly significant change from that, observed for wild-type controls (2.8) and MKO *cbs* ^+/^^−^ mice (3.0). Accumulation of AdoHcy has the potential to inhibit a wide range of cellular methylases, and recent work studying ethanol-induced liver injury found that relatively mild decreases in the hepatic AdoMet/AdoHcy ratio impair the phosphatidylethanolamine methyltransferase (PEMT)-mediated conversion of phosphatidylethanolamine to phosphatidylcholine, resulting in steatosis that could be reversed when the AdoMet/AdoHcy ratio was normalized by betaine treatment [18]. Although we only have data for the plasma levels of AdoMet and AdoHcy in the MKO and HO mice which may differ from hepatic levels, the level of decrease in AdoMet/AdoHcy ratio that we observed in the plasma of CBS deficient mice is approximately 7-fold greater than that reported in that study. It is therefore quite possible that there is some inhibition of PEMT in the MKO *cbs* null mice that has the potential to contribute to hepatopathy. In this context, it is interesting to note that the degree of hepatopathy in the MKO mouse model is very similar to that observed previously in the PEMT knockout mouse that incurs hepatic steatosis, which, when exacerbated by reduced dietary choline, results in severe liver injury and death [19].

While all of the above appear to be plausible contributory mechanisms for the hepatopathy observed in the MKO *cbs* null mice, it should be noted that the HO mice with essentially identical levels of tHcy, cysteine, methionine, AdoMet, and AdoHcy do not incur any hepatic steatosis or fibrosis. Presently, the only biochemical difference we have been able to identify between the MKO, TKO, and HO models is in the relative levels of cystathionine indicating that this compound may be exerting previously unsuspected hepatoprotective effects. To date, cystathionine has not been ascribed any physiological function other than serving as an intermediate in transsulfuration. However, a number of lines of evidence suggest that cystathionine may have previously unsuspected physiological roles. The observation that CBS but not CGL is expressed in a range of tissues during early development [20] and the apparent accumulation of cystathionine in adult human and primate brain [21] indicates that, in certain tissues and during early development, CBS is specifically involved in the production of cystathionine for reasons not related to cysteine synthesis. Previous work has suggested that the steatosis observed in MKO *cbs* null mice is directly related to the action of Hcy inducing endoplasmic reticulum (ER) stress and a consequent alteration of serum response element binding protein function resulting in dysfunctional lipid metabolism [22]. This possibility is consistent with the fact that our electron microscopy analysis found markedly distended ER in the liver samples of the MKO *cbs* null (see accompanying paper) but not in the HO mice (Fig. 5A) raising the possibility that cystathionine may be able to function as a chemical chaperone and thus block the induction of ER stress. Alternatively, a previous study using isolated hepatocytes and transfected *Xenopus* oocytes has indicated that thioether compounds such as methionine and cystathionine can act to block the induction of apoptosis by inhibiting the efflux of glutathione by up to 90% via a competitive interaction with the sinusoidal glutathione transporter [23]. The possible hepatoprotective effects of cystathionine are currently the focus of investigations in our laboratory.

Another transgenic mouse model of human CBS expression has been constructed where the human *CBS* gene is expressed under the control of a metallothionein promoter. Induction of this promoter results in significant production of human CBS protein that acts to lower tissue and plasma Hcy [24]. These findings prove that the human CBS protein can be functionally expressed in mice and results in active enzyme and indicates that the failure of our human *CBS* gene to express at normal levels is most probably due to our use of the human promoter. This group subsequently made a knock-in version of this mouse expressing the common pathogenic pyridoxine-responsive 1278T mutant also under the inducible control of the metallothionein promoter [25]. The I278T transgenic mouse was crossbred with *cbs* null mice to generate mice designated Tg-278Cbs−/− that expressed the human mutant gene and had no mouse CBS protein. These mice exhibited a significantly improved hepatic phenotype compared to *cbs* null mice and were rescued from neonatal lethality. It was found that the Tg-278Cbs−/− mice expressed low levels of CBS activity similar to those reported here for the HO mouse. Using an amino acid analyzer, this group reported average plasma levels of 250 μM Hcy and 72 μM methionine for these mice which are similar values to those reported here for the HO mouse. Unfortunately, this methodology was insufficiently sensitive to provide reliable data on cystathionine levels. Similarly, the levels of cysteine, AdoMet, and AdoHcy were not reported. The observation that very low level CBS activity was capable of improving the hepatic phenotype of *cbs* null mice and rescuing neonatal lethality without significantly lowering the extremely elevated Hcy is strikingly reminiscent of the HO mouse model. However, some differences between the Tg-278Cbs−/− mice and the HO model clearly exist. The Tg-278Cbs−/− mice were reported to incur facial alopecia, reduced body weight and some mild focal hepatic steatosis. [25]. None of these aspects were observed in the HO mice. It would be interesting to see if this mouse responds to betaine, exhibits a hypercoagulative phenotype, and exhibits elevated cystathionine.

The level of cystathionine observed in the HO mice is striking considering the very low level of CBS expression observed in this mouse model. To our knowledge, all genetically induced forms of elevated Hcy are associated with elevated cystathionine with the exception of HCU where cystathionine synthesis is largely abolished. In this paper, we report a number of lines of evidence indicating that the very high level of cystathionine accumulation in the HO mice is the result of severely elevated Hcy acting to impair CGL function. While it remains plausible that moderately elevated Hcy in conditions other than HCU is met by cystathionine accumulation due to increased CBS activity, the data presented in this paper offer a possible alternative explanation for the elevated cystathionine levels observed in other conditions where Hcy is elevated and transsulfuration is intact [9, 16].

An alternative explanation for the presence of elevated cystathionine in the HO mice is suggested by previous work studying the relative levels of methionine cycle metabolites in severe methionine adenosyltransferase I/III deficiency. This group found that in 13 patients with this condition, the patients with the most markedly elevated levels of plasma methionine also had elevations of plasma tHcy and mildly elevated plasma cystathionine. This study presented biochemical data indicating that methionine inhibits the activity of purified recombinant CGL [26]. A number of lines of evidence indicate that methionine is not responsible for the inhibition of CGL and subsequent accumulation of cystathionine in the HO mouse. Firstly, in the previous study, despite having very high levels of methionine (mean of 764 μmol/l (SD 459), normal reference range 13–45 μmol/l), the level of cystathionine accumulation in the methionine adenosyltransferase-deficient patients was very mild with the average value (329 nmol/l (SD 121)) being within the reference range for normal subjects (50–342 nmol/l), and the highest level detected in a methionine adenosyltransferase I/III-deficient patient was less than 58% higher than the upper limit of the normal reference range [26]. These patients presumably have normal levels of CBS activity and thus normal levels of cystathionine-generating capacity but exhibit significantly smaller elevations in cystathionine than the HO mice which have very low levels of CBS activity. Collectively, these observations indicate that the elevations of cystathionine seen in the HO mice are very unlikely to be occurring through the same mechanism as that seen in the human methionine adenosyltransferase I/III deficiency. It is worth noting that we also observed a small but statistically significant increase in plasma cystathionine levels in heterozygous *cbs*+/− MKO mice compared to their wild type littermates (*P* = 0.0233; Fig. 7A) with no elevation in plasma methionine levels. In addition, the MTHFR null mice showed a near three-fold increase in plasma cystathionine with no significant change in their plasma methionine levels (Fig. 8). While it remains possible that both methionine and Hcy are capable of impairing CGL function, our results indicate it is likely that the effect of Hcy is dominant in the HO mouse as lowering Hcy with betaine treatment clearly lowers plasma cystathionine levels while concomitantly increasing methionine (Fig. 8).

A recent study investigating the relative contributions of CBS and CGL to hydrogen sulfide synthesis via alternative transsulfuration reactions presented a mathematical model predicting that CGL might act to synthesize cystathionine directly from cysteine and Hcy *in vivo* and that this synthesis would be increased when Hcy was elevated to the levels typically observed in HCU. It was further postulated that elevated Hcy in homocystinuria due to remethylation defects would be accompanied by elevated cystathionine and a concomitant increase in cysteine synthesis [27]. If this assumption is correct, it would conceivably provide an alternative explanation for why we see elevated cystathionine in the MTHFR (−/−) mouse plasma and for the observation that cystathionine decreases in the HO mouse when Hcy is lowered by betaine treatment. However, a number of key facts argue against CGL-mediated synthesis being responsible for the elevated cystathionine that we have observed in the HO mice and the MTHFR deficient animals. Firstly, although we saw a statistically significant increase in cystathionine in both MKO *cbs* heterozygous mice and MTHFR null animals compared to their wild type littermate controls, none of these animals showed an increase in plasma cysteine levels. Similarly, in a previous work investigating Hcy, cystathionine, and cysteine levels in patients with pathogenic mutations in either Cbl C or Cbl D, both groups of patients showed elevated tHcy and cystathionine, but cysteine was either within the normal range or lower than normal [16]. Technical considerations associated with the determination of plasma cysteine levels mean that these parameters alone are probably not conclusive but perhaps the most convincing argument against a role for CGL in synthesizing detectable amounts of cystathionine *in vivo* comes from the plasma metabolite analysis of the HO and MKO mouse models. Both models have virtually identical levels of Hcy, AdoMet, methionine, and cysteine, but cystathionine is essentially undetectable in the MKO *cbs* (−/−) null model. Western blotting analysis of the MKO *cbs* null mice indicated that the level of hepatic CGL expression in these mice is essentially identical to that observed in the HO mice, indicating that this enzyme is not repressed in the MKO null mouse model and that even in the presence of very high levels of Hcy, CGL is not a significant source of cystathionine synthesis *in vivo* (data not shown)*.*

The ultimate aim of any animal model of a human disease is to recapitulate the major clinical sequelae without introducing any other defects that might interfere with the interpretation of experiments. The improved longevity and absence of severe liver injury in the HO mouse model of HCU is very advantageous and will allow future investigations into the pathophysiology of this disease without the possible interfering effects of fibrosis or altered lipid metabolism due to liver dysfunction. This paper represents the first time that a mouse model of HCU has been shown to exhibit a hypercoagulative phenotype. The fact that this hypercoagulative phenotype responds to treatment with betaine in a manner that is analogous to treatment of human patients suggests that future studies on thrombotic mechanisms in HO mice are likely to yield data that are relevant to the disease as it occurs in humans.
